# Novel *FOXG1* mutations in Chinese patients with Rett syndrome or Rett-like mental retardation

**DOI:** 10.1186/s12881-017-0455-y

**Published:** 2017-08-29

**Authors:** Qingping Zhang, Jiaping Wang, Jiarui Li, Xinhua Bao, Ying Zhao, Xiaoying Zhang, Liping Wei, Xiru Wu

**Affiliations:** 1 Department of Pediatrics, Peking University First Hospital, Beijing, 100034 China; 2 Center for Bioinformatics, State Key Laboratory of Protein and Plant Gene Research, School of Life Sciences, Peking University, Beijing, China

**Keywords:** *FOXG1*, RTT, RTT-like MR, Epilepsy, Hypoplasia of corpus callosum

## Abstract

**Background:**

We aimed to delineate clinical phenotypes associated with *FOXG1* mutations in Chinese patients with Rett syndrome (RTT) or RTT-like mental retardation (MR).

**Methods:**

Four hundred and fifty-one patients were recruited, including 418 with RTT and 33 with RTT-like MR. Gene mutations were identified by a target capture method and verified by Sanger sequencing.

**Results:**

Four *FOXG1* mutations were detected in four patients (three with RTT and one with RTT-like MR), including one previously described mutation and three *novel* mutations. These mutations included one missense and three micro-insertion mutations. Overall, 0.7% (3/418) of patients who had RTT in our cohort had *FOXG1* mutations. All patients had early global developmental delays followed later by severe mental retardation. None of the patients acquired speech or purposeful hand movements, and all of them presented with severe hypotonia, epilepsy, and hypoplasia of the corpus callosum.

**Conclusions:**

Our findings extend the spectrum of *FOXG1* mutations and the clinical features of RTT in Chinese patients. We recommend that patients with congenital RTT and Rett-like MR, especially those with brain malformations, such as hypoplasia of the corpus callosum, should be tested for *FOXG1* mutations.

## Background

Several Rett syndrome (RTT, OMIM #312750) variants have been described, including the classic, congenital, and early-onset seizure variants [1]. Mutations in the gene encoding methyl-CpG binding protein 2 (*MECP2*) cause 95% of cases of classical RTT and 40–50% of cases of atypical RTT, while the rate of gene mutation maybe varies among different ethnic variations [2]. Additionally, mutations in cyclin-dependent kinase-like 5 (*CDKL5,* NM_003159.2) have been identified in both females and males with the infantile seizure variant of RTT [3–5]. RTT was long thought to be an X-linked dominant condition because RTT is rare in males and both *MECP2* and *CDKL5* are located on the X chromosome. However, in 2008, the forkhead box protein G1 (*FOXG1,* NM_005249) gene located at 14q12 was found to be responsible for some cases of congenital RTT [6]. The congenital variant of RTT was initially described by Rolando in 1985 [7]. Patients with this disorder share the same clinical features as patients with classic RTT, but also suffer from hypotonia and mental retardation (MR) beginning in the first months of life.

*FOXG1* mutations have been identified in both female and male patients. The known spectrum of *FOXG1-*associated phenotypes includes atypical RTT, congenital RTT, a severe RTT-like neurodevelopmental disorder, and craniosynostosis [8–11]. Most patients with point mutations and deletions in *FOXG1* present with specific features, including generalized hypotonia, severe postnatal microcephaly, severe intellectual disability with an absence of speech, stereotypic movements, corpus callosum abnormalities, and seizures [8, 12, 13]. Larger deletions of 14q12 affecting *FOXG1* have been associated with dysmorphic facial features, whereas duplications of *FOXG1* result in developmental delay, intellectual disability with an absence of speech, infantile spasms, and an autism-like phenotype [14]. Heterozygous and somatic mosaic mutations in *FOXG1* can be pathogenic [13].

To our knowledge, Chinese patients with *FOXG1* mutations have not been reported to date. In this study, we report the identification of *FOXG1* mutations in four girls and describe their clinical symptoms. The purpose of this study was to improve the current understanding of the phenotypes associated with *FOXG1* mutations in Chinese RTT or RTT-like patients.

## Methods

### Patients

In total, 451 patients (433 females and 18 males) with RTT or RTT-like symptoms, including severe intellectual disability (ID) and autism-like behavior, were recruited from December 2012 to January 2016. Among them, 418 patients were identified as having RTT, of which 369 had the classic variant, 37 had the congenital variant, and 12 had the early-onset seizure variant. Seven male cases were included, of which six were classic RTT and the other was the congenital variant. The remaining 33 patients (22 females and 11 males) presented with RTT-like phenotypes, including microcephaly, stereotypic movements of the hands, and autism-like behaviors. Patients were evaluated for inclusion in this study based on a previously published set of clinical diagnostic criteria for RTT. Two points were given if the abnormality was severe, one if the abnormality was perceptible, but not extreme, and zero points were assigned if there was no abnormality [15]. A questionnaire asking for descriptions of clinical manifestations, electroencephalogram (EEG) results, magnetic resonance imaging (MRI) results, and family reproductive history was filled out by the parents of each patient. Patients were scored using a previously described scoring system [16].

### Gene mutational analysis

Genomic DNA was extracted using standard methods from the peripheral blood leukocytes of patients and their family members. The AmpliSeq library was prepared following the modified Ion AmpliSeq library preparation protocol (Pub No. MAN0006735). Ion AmpliSeq was used to perform a customized target region capture method based on multiplex PCR. Probes were designed to capture the regulatory regions of *MECP2* and the exon regions of *MECP2*, *CDKL5*, and *FOXG1* for mutation screening. The libraries were quantified by qPCR and pooled according to the molecular concentration. The pooled library was sequenced on the Illumina HiSeq 2500 system, generating approximately 1 M 100-bp paired end reads for each sample. The coverage of the third and fourth exon of the *MECP2* gene was 100%; the other coding regions of the three genes had coverages of greater than 92%. The average read depth for all samples was 780×, and the threshold depth was greater than 25 ×.

FastQC v0.10.1 was used to check the quality of reads and bwa0.7.12-r1039 was used to align the reads to the hg19 genome, producing a file in BAM format sorted by coordinates. Local realignment around indels and base quality score recombination by GATK v3.2 were performed using the bam file for pre-processing. Unified Genotyper and Haplotype Caller in GATK v.. 3.2 was used to call variants. Rare mutations whose population frequency was lower than 1% were filtered according to the 1000 Genomes data, ESP6500 population data, and ExAC data. Variants identified with target next-generation sequencing were confirmed by Sanger sequencing.

## Results

### Molecular analysis

**Fig. 1:**
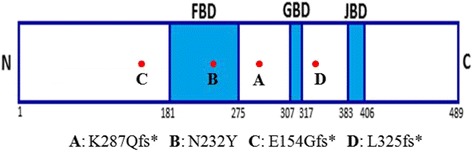
Schematic representation of the FOXG1 protein and patient mutations. The DNA binding fork-head domain (FHD), the Groucho-binding domain (GBD), and the JARID1B binding domain (JBD) of FOXG1 are shown. The numbers below the protein refer to the amino acid positions, and the mutations are indicated by dots

Mutations in *MECP2*, *CDKL5*, or *FOXG1* were found in 68.5% (309/451) of our patients, including 73.2% (270/369) of patients with the classic variant*,* 51.3% (19/37) of patients with the congenital variant*,* and 66.7% (8/12) of patients with the early-onset seizure variant. *FOXG1* gene mutations were identified in four female patients clinically classified as having congenital RTT or RTT-like MR. The *FOXG1* gene mutation rate was 0.7% (3/418) in RTT patients and 8.1% (3/37) in patients with congenital RTT. Three of the *FOXG1* mutations arose de novo in the patients*,* and we were unable to determine the origin in one case because a DNA sample was not available from the mother. Three patients had micro-insertion mutations. Patient 1 had a c.858dupC; p.K287Qfs*168 mutation, Patient 3 had a c.454dupG; p.E154Gfs*301 mutation, and Patient 4 had ac.972dupT; p.L325Ffs*130 mutation. Patient 2 had a missense mutation (c.694A > T; p.N232Y). Three of the mutations (those of patients 1, 2, and 4) in *FOXG1* were novel, i.e., they have not previously been described. A schematic representation of the FOXG1 protein, including the mutations identified here, is shown in Fig. 1.

### Patient clinical profiles and scores

The clinical and genetic data from the four patients with *FOXG1* mutations are summarized in Table 1. Patient 1 met the criteria for congenital RTT with a clinical score of 21. She was the second child of healthy non-consanguineous parents, and was born after an uneventful pregnancy. She developed epilepsy at 10 months of age and suffered from partial seizures accompanied by cyanosis. EEG at 3 months showed spikes and slow waves in the left anterior and posterior temporal lobes. At 1 year and 4 months of age, her EEG results were normal. MRI of the brain at 1 year showed atrophy of the cerebral hemisphere, delayed myelination in the central/periventricular white matter, and hypoplasia of the corpus callosum with underdevelopment of the frontal lobes. Her mother had previously lost a pregnancy, wherein embryonic development ceased after 2 months.

**Table 1:** Clinical Features of Subjects with FOXG1 mutations

	Case 1	Case 2	Case 3	Case 4
Mutation (AA change)	K287Qfs*168	N232Y	E154Gfs*301	L325Ffs*130
Age (m)	20	54	12	24
Head circumference (cm)	42	49	43	45.5
Rising head (m)	Y, 7 m	Y, 2 m	Y, 8 m	Y, 2 m
Sitting (m)	N	Y, 12 m	N	Y, 10 m
Walking	N	N, standing with acid at 24 m	N	N
Speech	N	N	N	N
Regression	Y	N	N	N
Stereotypic movements	Y, 12 m	Y, 10 m	Y, 3 m	Y, 8
Limited functional hand use	Y	Y	Y	Y
Bruxism	Y, 19 m	Y, 12 m	Y, 12 m	N
Hypotonia	Y	Y	Y	Y
Feeding difficulties	N	Y	Y	Y
Familial History	N	N	N	N
Sz Onset (m)	Y, 10.5	Y, 6	Y, 10	Y, 10.5
Sz types at Onset	Partial	Partial	Partial	Partial
Sz with cyanosis	Y	Y	Y	Y
Sleep disturbances	N	Y	Y	Y
EEG abnormalities	Y	Y	N	Y
Delayed myelination or hypomyelination	Y	N	N	N
Hypoplastic corpus callosum	Y	Y	Y	Y
Underdevelopment of frontal and temporal	Y	Y	Y	Y

Patient 2 did not fulfill the criteria for congenital RTT and was diagnosed with RTT-like MR. Pregnancy and birth were uneventful. Her developmental retardation was discovered at 4 months of age, when poor eye contact and eye tracking were observed. At 6 months of age, she began to have partial seizures accompanied by cyanosis. She had periodic swallowing problems that eventually resolved for unknown reasons. EEG at 1 year showed spikes and slow waves discharged in the central brain. MRI at 1 year and 4 months showed dysplasia of the corpus callosum. Another MRI at 1 year and 8 months showed bilateral enlargement of the lateral ventricles along with underdevelopment of the frontal lobes.

Patient 3 fulfilled the criteria for congenital RTT with a clinical score of 26. Developmental delays were noticed beginning at birth, including general weakness and poor eye contact. Epilepsy onset was at age 10 months, with partial seizures accompanied by cyanosis. EEG at 9 months was normal, but at 1 year and 7 months showed occasional atypical spikes and sharp spike discharges. A computed tomography (CT) scan and MRI of the brain at 4 months detected hypoplasia of the corpus callosum and underdevelopment of the frontal lobes. Her parents were both healthy.

Patient 4 fulfilled the criteria for congenital RTT with a clinical score of 24. She was the first child of the healthy parents. Abnormal muscular tension was noted at birth, and partial seizures with generalization and cyanosis began at the age of 10.5 months. At 1 year of age, the patient began to suffer from sleep disturbances and severe distress (crying). Eye contact and functional hand use were very poor from the time of birth. EEG at 1 year and 3 months showed multiple spikes and waves, and spikes and slow waves in the left occipital and posterior temporal regions. Mild underdevelopment of the frontal lobes was seen in a brain CT scan and MRI at the same age. Both of her parents were healthy and unrelated.

## Discussion

Our group has the largest Rett syndrome patient information database and specimen bank in China, and we recruited the largest number of Chinese RTT patients for *FOXG1* mutation screening reported in a single study to date. In this report, we detected four different *FOXG1* mutations in four female subjects, and three of these mutations were novel. These new patients provide support for delineating the clinical features of Chinese patients with *FOXG1* mutations. In addition, this is the first report of *FOXG1* mutations found in Chinese patients.

*FOXG1* plays an important role in the development of the telencephalon [17], and it contains only one coding exon that encodes the forkhead box protein G1 (FOXG1). FOXG1 is a DNA-binding transcription factor with a fork-head-binding domain that is only expressed in the fetal and adult brains and testis; it promotes cell proliferation in the telencephalon. Telencephalic progenitor cells differentiate prematurely in its absence, leading to early depletion of the progenitor population [18]. FOXG1 continues to be expressed in neurons postnatally and through adulthood. This expression profile might explain the particularly early onset of the neurological symptoms observed in the patients in this study. FOXG1 could also be one of several factors involved in oligodendrocyte precursor cell (OPC) proliferation and differentiation. As shown in Fig. 1, the FOXG1 protein consists of a forkhead DNA-binding domain (FHD; amino acids (AA) 181–275), a Gro-binding domain (GBD; AA 307–317), and a KDM5B (previously JARID1B) binding domain (JBD; AA 383–406). The FHD is highly conserved across all members of the FOX family. The GBD interacts with the Groucho protein, which is widely used by many developmentally important repressors for silencing target loci. The JBD recruits JARID1C demethylase, which is involved in demethylation of trimethylated and dimethylated Lys 4 of histone 3 (H3K4). Demethylation of H3K4 is associated with chromatin silencing [19].

*FOXG1*-related disorder is an autosomal dominant disorder. The position of a mutation in FOXG1 is expected to determine the extent of functional disruption [8]. Patient 1 had a mutation (p.K287Qfs*168) between the FHB and the GBD. Patient 2 had a missense mutation (p.N232Y) located within the highly evolutionarily conserved FHD of the protein that would likely impair DNA binding by FOXG1. Patient 3 harbored a *FOXG1* mutation (p.E154Gfs*301) that would generate a truncated protein lacking the FHD. Patient 4 had a single base insertion (p.L325Ffs*130) that would cause the loss of the JBD interaction domain and likely result in misfolding of the GBD [8]. The premature stop codons (PTCs) introduced by these mutations could trigger nonsense-mediated mRNA decay (NMD) of these transcripts in the fetal brain. Missense mutations or partial truncations of FOXG1 functional domains are expected to result in milder phenotypes owing to the partial retention of protein function [20]. However, Patient 2 had a missense mutation in the FHB domain and Patient 4 had a late truncation, and both presented with severe phenotypes similar to those of the other patients. Our results indicate that it is difficult to predict phenotypic severity based on the *FOXG1* mutation and suggest that additional factors contribute to the severity of *FOXG1*-related disorders, such as epigenetic and/or post-transcriptional regulation.

We observed the duplication of a guanine after a series of seven guanines in *FOXG1* (p.E154Gfs*301) in one of our patients, and this mutation has previously been reported in nine other patients [12, 14, 21]. These findings suggest that this region of the *FOXG1* gene is prone to replication errors and may represent a mutational hotspot. To the best of our knowledge, two patients whose parents have somatic mosaicism have previously been reported [13, 22]. In this study, three of the *FOXG1* mutations were de novo mutations, and the remaining one could not be classified owing to the lack of a maternal DNA sample. Although most *FOXG1* mutations arise de novo, parental mosaicism should be considered during genetic counseling as a risk factor for *FOXG1*-related disorders.

Our work confirms that *FOXG1* mutations can cause the congenital variant of RTT and RTT-like disorders. The shared features of the four patients with *FOXG1* mutations in our study were as follows: early global developmental delay and later severe mental retardation; hypotonia; failure to acquire speech or purposeful hand movements; deficient social interactions including poor eye contact, denoting a syndromic form of autism at an early age; combined stereotypic movements and dyskinesia with mixed features of athetosis, chorea, and dystonia; epilepsy with partial seizures accompanied by cyanosis; and hypoplasia of the corpus callosum along with underdevelopment of the frontal lobes. Other characteristic symptoms appeared only in some of the four patients, namely severe postnatal microcephaly (three patients), sleep disturbances (three patients), mood lability and inconsolable crying (two patients), developmental regression (one patient), and strabismus (one patient).

Compared with typical RTT patients, patients with *FOXG1* mutations were characterized by earlier developmental retardation, poorer language abilities, inability to walk, poorer development of the corpus callosum, underdevelopment of the frontal lobes, and a lack of a period of regression. There was no deterioration in three girls (Patients 2, 3, and 4), suggesting that the RTT definition may require expansion.

The results described in this report broaden the clinical spectrum of patient phenotypes associated with *FOXG1* mutations. Moreover, this is the first report of partial seizures accompanied by cyanosis in patients with *FOXG1* mutations. *FOXG1* mutations have been identified in both female and male patients [23]. Female patients with *FOXG1* mutations fit the classical RTT criteria, with either the congenital variant of RTT or an RTT-like neurodevelopmental disorder [14, 21]. Prior to 2016, more than 19 male patients with a *FOXG1* mutation or a chromosomal alteration affecting *FOXG1* had been reported, among which 13 presented with abnormal testis development (retractile testis) [11, 23]. These findings stress the importance of evaluating the *FOXG1* gene in females and males who present with the clinical features described above. They also highlight the potential importance of screening for *FOXG1* mutations in infants presenting with epilepsy.

Many patients with Rett syndrome or RTT-like disorders have been identified without known pathogenic mutations [24, 25]. Next-generation sequencing (NGS), whole-exome sequencing (WES), and array comparative genomic hybridization (aCGH) can be used to identify new causative genes and pathogenic mechanisms for Rett syndrome. However, there are no specific treatments that halt or reverse the progression of *FOXG1*-related disorders, such as congenital RTT. It is important to continue to improve the understanding of the consequences of *FOXG1* dysfunction to facilitate the development of novel therapeutic strategies.

## Conclusions

The *FOXG1* mutation screening described in this study involved the largest RTT patient cohort reported to date. The findings of this study expand the present understanding of the consequences of *FOXG1* dysfunction, and should help to inform the future development of therapeutic strategies. Based on our results, a lack of regression in patients with congenital RTT may indicate the involvement of *FOXG1* mutations, and therefore attention should be paid to whether the patient suffers regression. Overall, the results of this study suggest that screening for *FOXG1* mutations should be performed for patients with developmental features suggestive of congenital RTT, RTT-like MR, or brain malformations, such as hypoplasia of the corpus callosum accompanied by underdevelopment of the frontal lobes.

## Abbreviations

AA: amino acids
aCGH: array comparative genomic hybridization
CDKL5: cyclin-dependent kinase-like 5
CT: computed tomography
EEG: electroencephalogram
FHD: forkhead DNA-binding domain
FOXG1: forkhead box protein G1
GBD: Gro-binding domain
ID: intellectual disability
JBD: JARID1B binding domain
MECP2: Methyl-CpG binding protein 2
MR: Mental retardation
MRI: magnetic resonance imaging
NGS: Next-generation sequencing
NMD: nonsense-mediated mRNA decay
OPC: oligodendrocyte precursor cell
RTT: Rett syndrome
WES: whole-exome sequencing
